# A study on the effect of using the video teach-back method in continuous nursing care of stroke patients

**DOI:** 10.3389/fpubh.2024.1275447

**Published:** 2024-03-12

**Authors:** Fei Wang, Wen-Ming Feng, Ming Zhu, Qi Sun, Yong-Mei Zhang, Bing Wang, Xiao-Yong Luo, Jian-Tong Shen, Xiao-Wei Fang, Ting Zhang, Ge Cui

**Affiliations:** ^1^Department of Rehabilitation Medicine, The First People’s Hospital of Huzhou City, Huzhou City, Zhejiang, China; ^2^Department of General Surgery, The First People’s Hospital of Huzhou City, Huzhou City, Zhejiang, China; ^3^Department of Nephrology, The First People’s Hospital of Huzhou City, Huzhou City, Zhejiang, China; ^4^Huzhou University, Huzhou City, Zhejiang, China; ^5^Hospital Office, Qingchuan People's Hospital, Guangyuan City, China; ^6^Department of Pathology, The First People’s Hospital of Huzhou City, Huzhou City, Zhejiang, China

**Keywords:** stroke, video teach-back method, family caregiver, caring ability, continuous care

## Abstract

**Objective:**

To explore the effect of a video teach-back method on continuous family nursing care of stroke patients.

**Methods:**

Stroke patients hospitalized in our hospital between March 2020 and March 2023 who met the inclusion criteria were randomly divided into an intervention group (*n* = 45), who received routine health education plus video teach-back training of caregivers, and a control group (*n* = 45), who received routine health education only. The effects on nursing-related variables were compared between the two groups.

**Results:**

Total scores representing the caring ability of caregivers in the intervention group increased significantly over time relative to baseline and were higher than those of the control group. Scores representing the care burden of caregivers in the intervention group decreased significantly over time and were lower than those of the control group.

**Conclusion:**

The teach-back method combined with video education improves the nursing ability of family caregivers and can improve the self-care ability of stroke patients.

## 1 Introduction

Stroke is an acute-onset cerebral blood circulation disorder that is associated with high morbidity, high mortality, a high disability rate, a high recurrence rate, and many complications. The disease has become the primary cause of death and disability in middle-aged and older adults (1). With the growing older population and the rise in hypertensive patients, the incidence of stroke in China has increased (2).

At present, most stroke patients are transferred to their families after treatment alongside early rehabilitation in hospitals, and family caregivers such as spouses or children undertake the main care tasks (3, 4), which requires these caregivers to have sufficient professional knowledge (5). As such, the most-used approaches in foreign countries are the transitional care intervention model, generally delivered under a community health system, and the continuous nursing model, generally delivered by advanced practice nurses (6). Continuous care programs are designed on the basis of a series of actions to ensure that patients receive different levels of collaborative and continuous care in different health care settings and within the same health care facility (7). However, the provision of continuous care in China is afflicted by the problems of unbalanced regional development and insufficient human resources (8). The level of medical development in China is high in the east and low in the west, and it is difficult for nurses to carry out continuous nursing because of their intense daily work (9). Active care after discharge is critical for stroke patients, meaning that it is necessary to encourage patients’ families to participate in their management and to provide comprehensive rehabilitation care for them (10). Thus, China mainly promotes the continuous nursing model of hospital–community–family linkage. However, at present, family caregivers generally exhibit a lack of expertise and insufficient caring abilities (11). Previous studies have also found that mental and physical health is poorer among family caregivers than among the general population and that effective family care needs to be further explored (12). Medical staff should carry out health education for family caregivers before discharge of the patient, and provide them with information, skills, and emotional support pertaining to nursing care. This can improve the patient’s quality of life and quality of rehabilitation, thereby reducing the readmission rate and reducing the burden on family caregivers (13).

The teach-back method is an easy-to-understand, safe, and effective method of health education. The method involves the recipient (in this case, the family caregiver) expressing an understanding of the relevant information or skills in their own language after receiving health-related education; content that is wrong or has not been understood is then constantly re-emphasized until the caregiver correctly understands all the information (14). Video-based education involves the presentation of health education for patients or caregivers in the form of images, words, animations, and videos, which is conducive to ensuring concrete establishment of the health education content and the unification of standards, stimulating the interest of patients in learning, and helping patients to actively acquire disease-related knowledge and skills (15, 16). At present, video-based health education combined with the teach-back method has been applied under the model of continuous nursing and has achieved good results. Therefore, this study explored the impact of a video-based teaching method on continuous home care for stroke patients by training home caregivers via video-based teaching during the patient’s hospitalization.

## 2 Methods and materials

### 2.1 Study participants

A convenience sampling method was used to select stroke patients aged over 18 years who were diagnosed as having a first episode, were unable to take care of themselves fully (Brunnstrom’s stage I–III), and were conscious during computed tomography or magnetic resonance imaging examination of the head at our hospital between March 2020 and March 2023. These patients were then screened according to the inclusion and exclusion criteria for family caregivers. A total of 90 patients were included and were divided into an intervention group (*n* = 45) and a control group (*n* = 45) using the random number table method. The inclusion criteria for family caregivers were as follows: (1) age 18 or above; (2) immediate family member, in a stable physical condition of health and with normal understanding; (3) undertaking the main care tasks during hospitalization and continuing the main care work after discharge; (4) willing to participate in the study and able to cooperate with the investigator. The exclusion criteria for family caregivers were: (1) having a speech and/or language impairment or failing to communicate normally; (2) having received relevant training; and (3) participating in other clinical trials.

This study was approved by the Ethics Committee of The First People’s Hospital of Huzhou City (batch number: 2022KYLL002). All participants gave their informed consent and signed the consent form.

### 2.2 Study methods

#### 2.2.1 Preliminary preparation

A family caregiver training team was established; this comprised one physician, two experienced rehabilitation specialist nurses, one rehabilitation therapist, and two researchers (including the investigator), who were responsible for discussing and formulating the intervention program and determining the video content, as well as obtaining feedback and adjusting for any problems during the implementation process.

Regarding the development of the video content, the research team first discussed the development of an intervention program based on the existing literature and the previous rehabilitation experiences of stroke patients; this information was obtained via a search conducted using PubMed, CNKI, Web of Science, Embase, Scopus, and other databases. Subsequently, relevant experts were invited to evaluate and discuss the validity of the video content. We considered the opinions of the experts, and through repeated discussion within the research team, made a final determination as to the video content. After the video had been produced, we interviewed seven stroke patients and their family caregivers and invited them to watch part of the completed video in order to further modify and refine it according to their opinions and suggestions; based on this process, the final form of the video used for the current intervention was determined.

#### 2.2.2 Interventions

Routine health education was implemented in the control group. During the patient’s hospitalization, health education for family caregivers is mostly delivered orally or in writing by nurses or therapists. Routine health education for discharge mainly consists of daily life nursing and rehabilitation training of patients after discharge.

Both the intervention group and the control group received daily health education; the family caregivers in the intervention group received additional training via video-based teaching methods using the prepared videos. The research team aimed to select relevant videos for personalized intervention based on the needs of each family caregiver and the patient’s specific condition. To avoid contamination, family caregiver training was performed in a separate treatment room at the hospital. The first intervention was delivered after the study participants were enrolled, with the rehabilitation physician responsible for assessing the patients’ mental and physical condition to determine whether they could participate in the study. The specific content of the intervention included one course providing stroke-related information course, two courses on daily life nursing (including dietary care, dressing and undressing, etc.), and six courses on stroke rehabilitation training (including position transfer, balance training, walking training, etc.). The videos used in each training program were dependent on the patient’s condition and the needs of the family caregivers. Generally, stroke-related training was delivered first, and then a number of videos were selected from among the daily life nursing and stroke rehabilitation training options according to the specific circumstances of the patient and caregiver. Following the intervention, the videos were sent to or copied for the family caregivers so that they could watch and review them when necessary, especially after discharge.

### 2.3 Indicators and data collection

We collected baseline data for all patients and caregivers and subsequent score changes at 24 h after admission, 3 weeks after intervention, 7 weeks after discharge, and 3 months (15 weeks) after discharge.

A comprehensive care ability assessment questionnaire for stroke caregivers and the Zarit Burden Interview (ZBI) were used to evaluate the care ability and care burden of the caregivers in the two groups. The score on the Zarit scale is proportional to the caregiver burden. The stroke patient Motor Assessment Scale (MAS), the Barthel Index (BI), the Self-rating Anxiety Scale (SAS), and the Self-rating Depression Scale (SDS) were used to evaluate patients’ motor function, capacity for activities of daily living, and anxiety and depression in each of the two groups. Outcome measures were collected mainly via WeChat and telephone following discharge (for more details on the method, please refer to the Supplementary materials).

Cronbach’s α coefficient for the comprehensive care ability assessment questionnaire was 0.938, and the split-half reliability was 0.856. A total of 20 caregivers were randomly selected and the initial questionnaire was re-administered to this group two weeks after the first measurement. The test–retest reliability results showed that the test–retest correlation coefficient was 0.929, and the difference in results was statistically significant, *p* < 0.001.

### 2.4 Statistical analysis

Statistical analysis was performed using the SPSS 23.0 software package. Normally distributed data are reported in the form x ± s. Means were compared between multiple groups using one-way analysis of variance; pairwise comparisons between multiple groups were performed using Dunnett’s test; and repeated-measures analysis of variance was used for repeated-measures data. Non-normally distributed data are reported in the form *M* (Q1, Q3); inter-group comparisons for these variables were performed using the Kruskal–Wallis H rank sum test. Categorical data are expressed in the form of number of cases or percentages; *χ*^2^ tests were used for inter-group comparisons of these variables. A *p*-value of <0.05 was considered significant.

## 3 Results

### 3.1 Comparison of general variables

#### 3.1.1 Comparison of demographic variables for family caregivers

In this study, there were 45 family caregivers in each group; the mean age of the caregivers was 45.51 ± 9.83 years in the intervention group and 46.41 ± 10.24 years in the control group. Most patients in both groups were cared for by their spouses (71.11% vs. 64.44%), and the most common level of education among family caregivers in both groups was a secondary school education (42.22% vs. 48.89%). In addition, 46.67 and 53.33% of the families in the two groups, respectively, had a *per capita* monthly income of 3,001 yuan or more. There were no significant differences between the two groups in terms of age or gender of the family caregiver, their relationship with the patient, their level of education, or the *per capita* monthly household income (*p* > 0.05); the groups were comparable on all these variables, as shown in Table 1.

**Table 1:** Comparison of general family caregiver variables.

Basic information		Intervention group (*n* = 45)	Control group (*n* = 45)	*χ* ^2^ */t*	*p*
Gender (male/female)		23/22	20/25	0.401	0.527
Age (years, x ± s)		45.51 ± 9.83	46.41 ± 10.24	1.371	0.172
Relationship with patient (n)	Spouse	32	29	0.539	0.764
	Child	10	13		
	Other	3	3		
Education (n)	Primary school or below	7	8	4.053	0.256
	Middle school	19	22		
	University	11	13		
	Master’s degree or above	8	2		
Occupation (cases)	Clerk	13	15	3.324	0.344
	Self-employed	19	24		
	Retired	6	3		
	Other	7	3		
Monthly income *per capita* (RMB)	<1,000	4	3	0.986	0.805
	1,000 ~ 2000	9	6		
	2001 ~ 3,000	11	12		
	≥3,001	21	24		

#### 3.1.2 Comparison of demographic variables for patients

Comparison of the baseline data showed that the mean age of patients in the intervention group (*n* = 45) was 53.39 ± 12.51 years and that of patients in the control group (*n* = 45) was 54.71 ± 12.77 years. There were no significant differences between the two groups in gender composition ratio, age, smoking history, drinking history, body mass index, hypertension history, diabetes history, stroke type, Brunnstrom stage (hand, lower limbs, or upper limbs), or other indicators (*p* > 0.05), as shown in Table 2.

**Table 2:** Comparison of patient data between the two groups.

Basic information		Intervention group (*n* = 45)	Control group (*n* = 45)	*χ*^2^/*t*	*p*
Gender (male/female)		20/25	24/21	0.711	0.399
Age (years, x ± s)		53.39 ± 12.51	54.71 ± 12.77	1.371	0.172
Height (cm, x ± s)		159.32 ± 8.89	159.81 ± 11.94	1.122	0.305
Body mass index (kg/m^2^, x ± s)		19.52 ± 7.63	20.32 ± 8.75	0.798	0.440
Smoking history (n)		25	30	1.169	0.280
Alcohol history (n)		31	35	0.909	0.340
History of hypertension (n)		23	29	1.640	0.200
Hyperlipidemia (n)		16	17	0.048	0.827
Diabetes history (n)		9	13	0.963	0.327
Hyponatremia (n)		14	9	1.460	0.227
Low hemoglobin (n)		14	7	3.043	0.081
Hemiplegic side	Left	21	26	1.113	0.291
	Right	24	19		
Stroke type (n)	Ischemic stroke	21	25	0.711	0.399
	Hemorrhagic stroke	24	20		
Brunnstrom stage (hand)		26	29	0.522	0.914
		11	10		
		5	4		
		3	2		
Brunnstrom stage (lower limb)		25	29	0.747	0.688
		14	11		
		6	5		
Brunnstrom stage (upper limb)		31	28	0.443	0.801
		5	6		
		9	11		

#### 3.1.3 Comparison of baseline data between the two groups

To ensure the reliability of the study results, the baseline values on the evaluation indicators were compared between the two groups. The results showed that there were no statistically significant differences at baseline between family caregivers in the two groups in terms of total score for caring ability, scores on each of the four dimensions examined (namely, knowledge, skills, self-stress and health management, and coping strategies), or ZBI score (*p* > 0.05), meaning that the groups were comparable. Moreover, there were no statistically significant differences between the two groups at baseline in terms of MAS score, BI, SAS standard score, or SDS standard score (*p* > 0.05); these scores were also comparable, as shown in Table 3.

**Table 3:** Comparison of baseline scores between the two groups.

Item	Intervention group (*n* = 45)	Control group (*n* = 45)	*t*	*p*
Total nursing ability score (x ± s)	95.35 ± 12.13	96.24 ± 13.21	0.438	0.665
Knowledge score (x ± s)	16.67 ± 4.43	17.23 ± 3.67	1.382	0.180
Skill score (x ± s)	49.23 ± 4.45	48.34 ± 5.23	−0.569	0.574
Self-stress and health management score (x ± s)	16.56 ± 2.67	16.58 ± 2.21	0.839	0.401
Coping strategies score (x ± s)	15.34 ± 2.34	15.23 ± 2.94	0.132	0.896
ZBI score (x ± s)	39.23 ± 7.23	41.56 ± 7.34	−0.255	0.800
MAS score (x ± s)	20.45 ± 6.36	21.67 ± 6.43	1.423	0.110
BI score (x ± s)	43.19 ± 8.89	42.87 ± 9.78	0.055	0.956
SAS score (x ± s)	35.13 ± 4.87	35.92 ± 4.34	0.987	0.381
SDS score (x ± s)	36.23 ± 4.78	36.43 ± 3.23	1.122	0.556

ZBI, Zarit Burden Interview; MAS, Motor Assessment Scale; BI, Barthel Index; SAS, Self-rating Anxiety Scale; SDS, Self-Rating Depression Scale.

### 3.2 Comparison of the two groups on family caregivers’ caring ability and care burden

A one-way repeated-measures analysis of variance was conducted to investigate the effect of video teach-back training on the caring ability and care burden of the family caregivers. The Shapiro–Wilk test showed that the data for each group followed an approximately normal distribution (*p* > 0.05). Mauchly’s sphericity test indicated that differences were equal in the variance–covariance matrix for each group (*p* > 0.05); data are expressed in the form x ± s, as shown in Table 4. The results are summarized and described below.

**Table 4:** Comparison of caring ability and care burden between the caregivers in the two groups at baseline and at 3 weeks, 7 weeks, and 15 weeks after family caregiver training.

Item	Time	Intervention group (*n* = 90)	Control group (*n* = 100)	*F*_Interaction_/*P*_Interaction_ value	*F_Time_/P_Time_* value	*F_Treatment_/P_Treatment_* value
Total caregiving ability score	Baseline	95.35 ± 12.13	96.24 ± 13.21	321.132/0.001	119.723/0.001	342.734/0.001
	3 weeks	100.23 ± 11.34	97.45 ± 11.23			
	7 weeks	105.84 ± 10.85	98.86 ± 11.54			
	15 weeks	110.54 ± 12.92	103.23 ± 10.65			
Knowledge	Baseline	16.67 ± 4.43	17.23 ± 3.67	231.322/0.001	211.251/0.001	256.321/0.001
	3 weeks	21.45 ± 3.12	19.54 ± 3.02			
	7 weeks	22.22 ± 2.33	21.23 ± 3.19			
	15 weeks	25.54 ± 2.12	21.31 ± 3.11			
Skills	Baseline	49.23 ± 4.45	48.34 ± 5.23	165.211/0.001	411.723/0.001	321.734/0.001
	3 weeks	52.23 ± 4.33	48.65 ± 4.56			
	7 weeks	53.86 ± 4.72	47.32 ± 4.73			
	15 weeks	55.19 ± 4.94	48.13 ± 5.33			
Self-stress and health management	Baseline	16.56 ± 2.67	16.48 ± 2.21	422.862/0.001	289.451/0.001	311.661/0.001
	3 weeks	17.33 ± 1.57	16.56 ± 2.34			
	7 weeks	19.32 ± 1.89	16.33 ± 2.22			
	15 weeks	20.58 ± 1.24	17.16 ± 2.19			
Coping strategies	Baseline	15.34 ± 1.34	15.23 ± 2.94	426.443/0.001	324.451/0.001	544.123/0.001
	3 weeks	16.28 ± 1.92	16.34 ± 2.34			
	7 weeks	18.12 ± 1.78	16.78 ± 2.65			
	15 weeks	20.52 ± 1.96	17.24 ± 2.57			
ZBI score	Baseline	39.23 ± 7.23	41.56 ± 7.34	322.978/0.001	311.539/0.001	424.447/0.001
	3 weeks	37.23 ± 4.67	39.64 ± 5.18			
	7 weeks	34.46 ± 5.56	37.59 ± 4.34			
	15 weeks	31.98 ± 6.43	35.77 ± 4.67			

ZBI, Zarit Burden Interview.

#### 3.2.1 Comparison of caring ability between the two groups

In terms of caregiving ability: The total score of care ability and the scores of the four sub dimensions of two groups of family caregivers were was significant (*p* < 0.001), indicating that the changes over time (across the four time points) in total caregiver ability score, knowledge dimension score, and skill dimension score differed between the two groups. In addition, the scores on each index increased over time in both groups (*p* < 0.001), indicating that carers’ overall caring ability and ability on each of the four dimensions improved significantly over time. Finally, the different training methods used in each of the two groups had different effects on total caring ability score and scores on each of the four dimensions (*p* < 0.001), with all scores being higher for caregivers in the intervention group than for those in the control group.

#### 3.2.2 Comparison of care burden between the two groups

In terms of care burden, the interaction between time and group was significant (*p* < 0.001) in the effect on caregiver burden score, indicating that there were differences between the two groups in the change in score over time. ZBI scores decreased over time in both groups (*p* < 0.001), indicating that the caregivers’ care burden scores were reduced significantly over time. Moreover, the different training methods used in each group had an effect on caregiver burden scores(*p* < 0.001), with scores being lower in the intervention group than in the control group.

### 3.3 Comparison of patient scores between the two groups

A one-way repeated-measures analysis of variance was used to investigate the effect of video teach-back training of family caregivers on patient outcomes. The Shapiro–Wilk test showed that the data for each group followed an approximately normal distribution (*p* > 0.05). As shown in Table 5, Mauchly’s sphericity test showed that differences were equal in the variance–covariance matrix (*p* > 0.05) for each group; data are expressed in the form x ± s, and the results can be summarized and described as follows. The interaction between group and time was significant in the effect on MAS score, BI score, SAS score, and SDS score (*p* < 0.001), indicating that the changes in these variables over time differed between the two groups. In addition, scores on each of these indices increased over time in both groups (*p* < 0.001), indicating that the patients’ condition on these dimensions changed significantly over time. Finally, the different training methods had different effects on MAS score, BI score, SAS score, and SDS score (*p* < 0.001), with MAS and BI scores being higher among patients in the intervention group than those in the control group, and SAS and SDS scores being lower among patients in the intervention group than those in the control group.

**Table 5:** Comparison of patient MAS/BI/SAS/SDS scores between the two groups at baseline and at 3 weeks, 7 weeks, and 15 weeks after training.

Item	Time	Intervention group (*n* = 90)	Control group (*n* = 100)	*F_Interaction_/P_Interaction_* value	*F_Time_/P_Time_* value	*F_Treatment_/P_Treatment_* value
MAS score	Baseline	20.45 ± 6.36	21.67 ± 6.43	1.387/0.219	423.224/0.001	225.321/0.001
	3 weeks	25.44 ± 5.89	23.55 ± 4.76			
	7 weeks	32.66 ± 6.78	29.39 ± 6.35			
	15 weeks	36.56 ± 6.32	32.17 ± 4.83			
BI score	Baseline	43.19 ± 8.89	42.87 ± 9.78	2.782/0.102	421.123/0.001	314.312/0.001
	3 weeks	56.38 ± 5.39	55.82 ± 4.23			
	7 weeks	66.29 ± 4.72	62.39 ± 5.19			
	15 weeks	75.33 ± 6.72	66.35 ± 7.34			
SAS score	Baseline	35.13 ± 4.87	35.92 ± 4.34	1.652/0.203	235.234/0.001	421.642/0.001
	3 weeks	34.67 ± 4.53	35.10 ± 5.22			
	7 weeks	32.21 ± 5.56	34.69 ± 3.98			
	15 weeks	29.45 ± 4.97	33.45 ± 4.56			
SDS score	Baseline	36.23 ± 4.78	36.43 ± 3.23	1.862/0.163	312.141/0.001	423.534/0.001
	3 weeks	35.41 ± 3.67	36.34 ± 2.98			
	7 weeks	33.54 ± 4.56	34.19 ± 3.87			
	15 weeks	30.87 ± 3.56	32.34 ± 3.21			

MAS, Motor Assessment Scale; BI, Barthel Index; SAS, Self-rating Anxiety Scale; SDS, Self-rating Depression Scale.

## 4 Discussion

Stroke has always been among the most important cardiovascular and cerebrovascular diseases in China. In this study, we found that the teach-back method is an easy-to-understand, safe, and effective method of health education (13). Normative family continuity care helps patients and their families to receive targeted healthcare guidance and plays an important role in promoting recovery from disease (17, 18).

For family caregivers, the results of this study showed that there were significant effects of time, group, and the time–group interaction on total caring ability scores and scores on each of the four dimensions of caring abilities. Moreover, caregivers’ total caring ability scores, scores on the knowledge dimension, and scores on the skill dimension significantly increased over time relative to baseline in the intervention group. This shows that the use of the video teach-back method to train family caregivers significantly improves their practical skills and knowledge levels. This is consistent with the findings of previous studies, in one of which, eight health education videos related to positioning, movement, and transfer of subacute stroke patients were developed and health education was delivered to informal caregivers at the patients’ homes for 3 days, thereby improving the practical skills and knowledge of the caregivers (19). Additionally, the dimensions of both ‘self-stress and health management’ and ‘coping strategies’ also presented the same results as the aforementioned dimensions, which is also consistent with previous findings. One study included 300 stroke patients and their caregivers, who received training on 3–5 occasions for 30–45 min during their hospitalization; it was found that caregivers who participated in this intervention experienced a significantly reduced caregiver burden, less anxiety and depression, and a higher quality of life after the intervention (20).

In terms of patients, the results of this study showed that there were significant effects of time, group, and the time–group interaction on MAS score, BI score, SAS score, and SDS score. Moreover, the MAS and BI scores of patients in the intervention group increased significantly over time relative to the baseline, and were higher than those of patients in the control group (*p* < 0.05). This demonstrates that the use of the video teach-back method to train family caregivers could significantly improve motor function and activities of daily living in stroke patients at 7 and 15 weeks after intervention, and that this effect is time-dependent; this indicates that this intervention has a positive effect on the rehabilitation of stroke patients after discharge.

According to the cognitive theory of multimedia learning, video-based health education can enhance the effects of health education compared with written or oral health education methods (21). In the present study, video health education was combined with the teach-back method, providing a two-way learning process and further promoting family caregivers’ mastery of relevant rehabilitation knowledge and skills. In terms of psychological status, both the SAS scores and the SDS scores of the patients in the intervention group decreased significantly over time relative to the baseline and were lower than those of patients in the control group (*p* < 0.05). This finding is in agreement with those of Zhang et al. (22), who demonstrated that health education programs for caregivers of ischemic stroke patients significantly reduced anxiety and depression scores and cognitive dysfunction among patients. An important reason for this improvement in anxiety and depression may be that it is directly related to the improvement in motor function.

This study also has some limitations. First, due to time and resource constraints, the sample size was small and the representativeness of the sample may be low, meaning that further exploration is needed via large-sample, multicenter studies. Furthermore, this study included insufficient observations of patients receiving educational programs and undergoing rehabilitation exercises, which can be further rectified in future studies.

In summary, the teach-back method combined with video-based education combines the advantages of the bidirectional feedback of the former and the simple and quick video education of the latter to improve the care abilities of family caregivers and effectively reduce their care burden. At the same time, this method can improve motor function and activities of daily living among stroke patients, as well as their levels of anxiety and depression.

## Data availability statement

The original contributions presented in the study are included in the article/Supplementary material, further inquiries can be directed to the corresponding author.

## Ethical statement

The studies involving humans were approved by the First People’s Hospital of Huzhou City (ethical batch number: 2022KYLL002). The studies were conducted in accordance with the local legislation and institutional requirements. The participants provided their written informed consent to participate in this study.

## Author contributions

FW: Conceptualization, Data curation, Investigation, Methodology, Project administration, Resources, Software, Supervision, Validation, Visualization, Writing – original draft, Writing – review & editing. W-MF: Conceptualization, Funding acquisition, Investigation, Methodology, Resources, Supervision, Visualization, Writing – original draft, Writing – review & editing. MZ: Conceptualization, Data curation, Investigation, Methodology, Project administration, Resources, Software, Supervision, Validation, Visualization, Writing – original draft, Writing – review & editing. QS: Conceptualization, Investigation, Methodology, Project administration, Resources, Software, Validation, Visualization, Writing – original draft, Writing – review & editing. Y-MZ: Conceptualization, Formal analysis, Investigation, Methodology, Project administration, Supervision, Visualization, Writing – original draft, Writing – review & editing. BW: Conceptualization, Investigation, Methodology, Resources, Supervision, Writing – original draft, Writing – review & editing. X-YL: Conceptualization, Investigation, Methodology, Project administration, Resources, Validation, Writing – original draft, Writing – review & editing. J-TS: Formal analysis, Investigation, Methodology, Software, Validation, Writing – original draft, Writing – review & editing. X-WF: Conceptualization, Data curation, Investigation, Methodology, Validation, Writing – original draft, Writing – review & editing. TZ: Investigation, Methodology, Project administration, Validation, Writing – review & editing. GC: Investigation, Methodology, Resources, Visualization, Writing – review & editing.
