# Supplementary material for: A study on the effect of using the video teach-back method in continuous nursing care of stroke patients
Source: Front Public Health. 2024 Mar 12;12:1275447. doi: 10.3389/fpubh.2024.1275447 (PMC10964721; doi:10.3389/fpubh.2024.1275447)
Supplement: Supplementary file 1 [file Data_Sheet_1.docx]

Stroke Caregiver Comprehensive Care Ability Assessment Questionnaire

Instructions: The following are questions about stroke knowledge. Please use ' √ ' to make a choice between ' don 't know ', ' know some ', ' basically know ' and ' completely know ' according to your understanding of these questions.

| Content | not know | Know some | Basically know | fully know |
| --- | --- | --- | --- | --- |
| 1.I know what factors will increase the risk of stroke. |  |  |  |  |
| 2.I know what prodromal symptoms patients usually have before stroke. |  |  |  |  |
| 3.I know what sequelae stroke will bring to patients. |  |  |  |  |
| 4.I know how to guide patients to carry out rehabilitation exercises to promote their functional recovery. |  |  |  |  |
| 5.I know what stroke patients should pay attention to diet. |  |  |  |  |
| 6.I know the role of drugs taken by patients and the possible side effects. |  |  |  |  |
| 7.I know how to prevent complications (such as aspiration pneumonia, pressure sores, urinary tract infections, etc.) in patients with long-term bedridden stroke. |  |  |  |  |
| 8.I know how to prevent the recurrence of stroke. |  |  |  |  |
| 9.When stroke patients relapse, I know how to deal with. |  |  |  |  |

The following is about the problems that you may encounter in your daily care work. According to your actual situation, use ', ' not able to ', ' slightly able to ', ' basically able to ', ' fully able to ' to make a choice.

| Content | not able to | slightly able to | basically able to | fully able to |
| --- | --- | --- | --- | --- |
| 10.I can easily cope with the daily life of the patient (such as helping with diet, wearing and undressing, toileting, bathing, position transfer, etc.). |  |  |  |  |
| 11.I can selectively help patients to complete some daily activities according to their daily activities, rather than assisting them all. |  |  |  |  |
| 12.I was able to guide, supervise and assist patients with rehabilitation exercises. |  |  |  |  |
| 13.I can guide patients to take medicine correctly according to the doctor's advice. |  |  |  |  |
| 14.I can determine which symptoms of the patient may be caused by the drug. |  |  |  |  |
| 15.I can urge patients to change some bad habits (such as smoking, drinking, high salt and high fat diet, etc.). |  |  |  |  |
| 16.I can monitor the patient 's physical condition (such as blood pressure, blood glucose, activity, etc.), and notice the slight changes in their condition. |  |  |  |  |
| 17.When the patient has discomfort symptoms, I can judge whether it is a recurrence of stroke. |  |  |  |  |
| 18.If the patient relapses, I can deal with it calmly and freely. |  |  |  |  |
| 19.I can keenly detect the patient's emotional changes. |  |  |  |  |
| 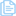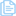20.When a patient is depressed and pessimistic, I can always make him (her) feel better in various ways. |  |  |  |  |
| 21.I can understand the needs of patients through their verbal or nonverbal behavior. |  |  |  |  |
| 22. I am able to meet the needs of patients (such as physical, psychological, social activities, etc.) |  |  |  |  |
| 23, I can according to the patient 's condition changes, rehabilitation, to constantly adjust their care work (such as diet adjustment, rehabilitation exercise, etc.) |  |  |  |  |
| 24.When there are many problems in the process of taking care of patients, I can deal with them in an orderly manner according to the priority of the problem. |  |  |  |  |
| 25.In the process of caring for patients, I can communicate with patients, respect their opinions, and let them participate in the care work. |  |  |  |  |
| 26.When negative emotions (such as irritability, anger, sadness) occur due to caring for patients, I can adjust my emotions in time. |  |  |  |  |
| 27.When I feel physically and mentally exhausted from caring for patients, I can relieve myself in some ways. |  |  |  |  |
| 28.In meeting the needs of patients, I am also able to meet my own needs (such as rest, entertainment, social activities, etc.). |  |  |  |  |
| 29.I can always maintain a positive and optimistic attitude to take care of patients. |  |  |  |  |
| 30.While taking care of patients, I can also take good care of myself. |  |  |  |  |
| 31.In the process of caring for patients, I can also actively seek relevant knowledge and information to solve the care problems encountered. |  |  |  |  |
| 32.I am able to take a critical view of all the information I receive about illness and care through various channels, rather than accept it as a whole. |  |  |  |  |
| 33.I was able to communicate in a timely manner with other members of the family to work together on the care issues I encountered. |  |  |  |  |
| 34.In the process of taking care of patients, I was able to take the initiative to ask for help from relatives. |  |  |  |  |
| 35.In the process of caring for patients, I can take the initiative to ask for help from relatives35. I can make full use of other social resources to solve the care problems I encounter. |  |  |  |  |
