# Supplementary material for: A study on the effect of using the video teach-back method in continuous nursing care of stroke patients
Source: Front Public Health. 2024 Mar 12;12:1275447. doi: 10.3389/fpubh.2024.1275447 (PMC10964721; doi:10.3389/fpubh.2024.1275447)
Supplement: Supplementary file 2 [file Data_Sheet_2.docx]

1. Study methods

1.1 Preliminary preparation

1.1.1 Team members division of labor

After establishment of training team, among them, the intervention protocol and video content were jointly discussed and determined by the research team. Rehabilitation specialist nurses, rehabilitation therapists and researchers are jointly responsible for video shooting and production. During the implementation, the rehabilitation physician is responsible for assessing the patient 's condition and physical condition, determining whether he/she can participate in the study, and working with the rehabilitation therapist to develop a personalized rehabilitation plan for the patient, and timely updating the rehabilitation plan according to the patient's condition and rehabilitation status. Rehabilitation specialist nurses and researchers were responsible for recruiting participants, screening hospitalized stroke patients, including patients who met the inclusion criteria and their family caregivers, and training family caregivers. Before training family caregivers, in stroke rehabilitation training, rehabilitation specialist nurses and researchers uniformly received training from rehabilitation therapists to ensure standardization and consistency of rehabilitation training movements. Outcome measures were measured and data analyzed by another researcher blinded to participant group assignment.

1.1.2 Preparation of video content

As experts’ suggestions to add a video item "How to prevent and cope with stroke recurrence". In rehabilitation training, it is recommended to delete the video item of "good limb placement", and add the video of muscle stretching at the commonly affected site in addition to the basic rehabilitation movements. In terms of video production, because this study trained mainly family caregivers of stroke patients, it is recommended to add instructive language and pictures to the video to help family caregivers master the relevant knowledge and learn to do rehabilitation training and so on. We comprehensively consider the opinions of all experts and modify them through repeated discussions with the research team, and finally determine that the video content includes three major aspects: stroke-related knowledge, daily life care, and rehabilitation training, while covering what is stroke, identification of stroke and secondary prevention, wearing clothing, diet care, postural transfer, and balance training. Next, we look up the existing video resources of the hospital and identify the video content that needs to be shot and recorded by ourselves, which mainly focuses on rehabilitation training.

Video shooting scripts were then co-written by researchers, rehabilitation therapists, and nurses based on established health education video content and filmed under the guidance and supervision of professional video photographers. After video production, before the intervention, to assess the face validity of the video, we interviewed seven stroke patients and their family caregivers and invited them to randomly watch some of the completed videos to understand their opinions and suggestions on the video, and then we further modified and refined the video according to the participants' opinions and suggestions. Eventually formed the video library currently used for the intervention.

1.2 Interventions

1.2.1 Health education in the control group

Routine health education mainly includes 3 aspects: 1. disease-related knowledge is mainly to inform family caregivers of stroke-related knowledge by nurses during hospitalization (such as what is stroke, stroke identification and secondary prevention, etc.); 2. daily life nursing related health education content includes informing family caregivers of different stages of disease nursing points and rehabilitation nursing precautions (such as diet nursing, disease observation, wearing off clothing, etc.); 3. rehabilitation training related health education content is mainly informed by therapists how family caregivers help patients to carry out rehabilitation exercise and rehabilitation exercise precautions, teaching caregivers of rehabilitation exercise related movements, etc. Routine discharge health education was also given to patients at discharge.

1.2.2 The training detail on the intervention group

The hospitalization cycle of patients in the recruited hospitals was 3 weeks, the duration of training for caregivers was 3 weeks, and the length and frequency of training were 30 minutes each time, 3 times a week. Specific content includes one stroke related knowledge course, two daily life nursing courses (including diet nursing, dressing and undressing, etc.), and six stroke rehabilitation training courses (including postural transfer, balance training, walking training, etc.).Specifically, the videos used for each training were based on the patient 's condition and the care needs of family caregivers, and training on stroke-related knowledge was generally performed first, and then some videos were selected from daily life care and stroke rehabilitation training according to the specific circumstances of patients and caregivers for training. The implementation process of each course was mainly performed according to four steps of the teach-back method: (1) Interpretation: Patients and caregivers were informed of the main content and significance of the training before the intervention, their consent was obtained, and then researchers and participants watched the video together. (2) Assessment: After video playback, family caregivers' understanding and mastery of the knowledge or skills taught were assessed, and open-ended questions were used according to the video content, such as "What are the risk factors leading to stroke?" How to help patients dress, can you do it over and over? ". (3) Clarification: For the answers or demonstrations of caregivers, timely praise and praise shall be given for the correct places, and timely clarification and correction shall be given for the incorrect or irregular places, and the video shall be replayed or demonstration and explanation shall be given when necessary. (4) Understanding: Caregivers were again assessed for specific content until they fully understood or correctly mastered the relevant knowledge.

1.3 Data collection

Stroke caregiver comprehensive care ability assessment questionnaire, used to reflect stroke caregivers' comprehensive care ability ^[1]^, uses Liket-4 level scoring method, with a score of 1-4 points. The questionnaire consists of two parts and four dimensions: one is the degree of knowledge about stroke, which is divided into four levels from never knowing to fully guiding. The other is the evaluation of three dimensions of caregiver care skills, self-stress and health management, and coping strategies, which are never able, slightly able, basically able, and completely able to be divided into four levels.

The Zarit Caregiving Burden Scale consists of 22 items, each scored from 0 to 4, followed by none, occasional, sometimes, often, always, giving a total score of 88, with higher scores representing greater caregiver burden.

The assessment scale for motor function in stroke patients was proposed by Carr JH et al. ^[2]^ in 1985 and is mainly used to assess motor function in stroke patients. The scale has 9 dimensions, each comprising 7 levels and 6 actions, with scores ranging from 0 to 6. Eight of these dimensions were related to motor function, and the evaluation included supine to healthy lateral decubitus, supine to bedside sitting, sitting balance, sitting to standing, walking, upper limb function, and fine hand movements. An additional dimension is associated with total body muscle tone, which is not included in the total score ^[3]^.

Barthel Index is used to assess patients' activities of daily living. It mainly includes 10 items: eating, bathing, grooming, dressing, stool control, urination control, toilet use, bed chair transfer, flat ground movement, and ascending stairs. Each item is scored on a scale of 0-15, with a full score of 100 points, with higher scores indicating greater independence. Patients with scores between 80 and 100 were considered independent in terms of activities of daily living, patients with 61-79 needed assistance in activities of daily living, patients with 40-60 were partially dependent, patients with 20-39 were very dependent, and patients with less than 20 were completely dependent ^[4]^. It has good inter-observer reliability, with kappa statistics between 0.47 and 1.00, and intra-observer reliability, with kappa statistics between 0.84 and 0.97^[5]^.

The self-rating anxiety scale was developed by Zung et al. ^[6]^ in 1971 to evaluate patients' anxiety. The scale is a patient checklist with 20 items that are scored on a 4-point scale, with "1" indicating no or little time; "2" indicating sometimes; "3" indicating most time; and "4" indicating most time, with 5 items being reverse scored. The crude score was obtained by adding the 20 scores, and the standard score was obtained by taking the whole after the crude score was 1.25. The higher the score, the more severe the anxiety of the patients. The scale has been widely used to evaluate the psychological status of stroke patients and has demonstrated good reliability and validity.

The self-rating depression scale was developed by Zung et al. ^[7]^ in 1965 as a patient self-rating scale to evaluate a patient 's depressive mood. There are 20 items in the scale, including 10 forward items and 10 reverse items, using a 4-level score, "1" indicates no or little time; "2" indicates sometimes; "3" indicates most time; "4" indicates most time, adding the 20 scores to obtain a coarse score, and taking the whole after the coarse score of 1.25 to obtain a standard score, the higher the score, the more serious.

Reference:

[1] Wang ZL, Zhao Y. Development and Evaluation of Comprehensive Caregiving Ability Assessment Questionnaire for Stroke Caregivers. Chinese Journal of Practical Nursing. 2012;(19):75-77.

[2] Carr JH, Shepherd RB, Nordholm L, Lynne D. Investigation of a new motor assessment scale for stroke patients. Phys Ther. 1985;65(2):175-180. doi:10.1093/ptj/65.2.175.

[3] Johnson L, Selfe J. Measurement of mobility following stroke: a comparison of the Modified Rivermead Mobility Index and the Motor Assessment Scale. Physiotherapy. 2004;90(3):132-138. doi: 10.1016/j.physio.2004.01.004.

[4] Granger CV, Dewis LS, Peters NC, Sherwood CC, Barrett JE. Stroke rehabilitation: analysis of repeated Barthel index measures. Arch Phys Med Rehabil. 1979;60(1):14-17.

[5] Cid-Ruzafa J, Damián-Moreno J. Valoración de la discapacidad física: el indice de Barthel [Disability evaluation: Barthel's index] [published correction appears in Rev Esp Salud Publica 1997 Jul-Aug;71(4):411]. Rev Esp Salud Publica. 1997;71(2):127-137.

[6] Zung WW. A rating instrument for anxiety disorders. Psychosomatics. 1971;12(6):371-379. doi:10.1016/S0033-3182(71)71479-0.

[7] ZUNG WW. A SELF-RATING DEPRESSION SCALE. Arch Gen Psychiatry. 1965; 12:63-70. doi:10.1001/archpsyc.1965.01720310065008.
